# Construction and validation of a predictive nomogram model for invasive fungal infections in sepsis patients with severe pneumonia in the ICU

**DOI:** 10.3389/fcimb.2026.1854135

**Published:** 2026-07-01

**Authors:** Qi Xin, Longyang Ma, Xiaoyuan Yu, Gongliang Du

**Affiliations:** 1 Department of Emergency Surgery, Shaanxi Provincial People’s Hospital, Xi’an, China; 2 Department of Hematology, The Affiliated Hospital of Northwest University, Xi’an No. 3 Hospital, Shaanxi, Xi’an, China

**Keywords:** invasive fungal infection, nomogram, prediction model, sepsis, severe pneumonia

## Abstract

**Background:**

Invasive fungal infections (IFI) represent a serious complication in critically ill sepsis patients with severe pneumonia, contributing to increased mortality and prolonged hospitalization. Early prediction of IFI remains challenging due to the lack of specific clinical tools. This study aimed to develop and validate a predictive nomogram for IFI risk in this high-risk population.

**Methods:**

A total of 1,890 sepsis patients with severe pneumonia admitted to the ICU of the primary center were retrospectively enrolled from Shaanxi Provincial People’s Hospital. Patients were randomly divided into a training set (n = 1,418) and an internal validation set (n = 472). Additionally, an independent external validation cohort of 378 patients from another tertiary hospital (Xi’an No. 3 Hospital) was collected. Least Absolute Shrinkage and Selection Operator (LASSO) regression and multivariate logistic regression were used to identify independent predictors. A nomogram was constructed and evaluated for discrimination, calibration, and clinical utility using receiver operating characteristic (ROC) curves, calibration plots, and decision curve analysis (DCA).

**Results:**

The incidence of IFI was 25.8% (488/1,890). Eight independent predictors were identified: respiratory rate (RR), diabetes, respiratory failure (RF), acute-on-chronic liver failure (ACFL), prothrombin activity (PTA), D-dimer, activated partial thromboplastin time (APTT), and lactate. The nomogram demonstrated excellent discrimination, with area under the curve (AUC) values of 0.859 (training), 0.830 (internal validation), and 0.872 (external validation), outperforming the Sequential Organ Failure Assessment (SOFA) score. Calibration and DCA confirmed its clinical applicability.

**Conclusion:**

We developed and validated an easy-to-use nomogram that accurately predicts the risk of IFI in sepsis patients with severe pneumonia. This tool may assist clinicians in early identification and intervention for high-risk individuals.

## 1 Introduction

Sepsis is a life-threatening organ dysfunction caused by a dysregulated host response to infection, and it represents a major global health burden with an estimated incidence of 48.9 million cases and 11 million sepsis-related deaths annually (Rudd et al., 2020). Severe pneumonia is one of the most common primary infections leading to sepsis and ICU admission, accounting for approximately 30-50% of all sepsis cases (Rhee et al., 2017; Fleischmann-Struzek et al., 2020). In this critically ill population, the risk of secondary infections is substantially heightened, with invasive fungal infections (IFI) posing a particularly severe threat. The incidence of IFI patients, such as those with sepsis and severe pneumonia, has been steadily increasing. Recent epidemiological studies report that Candida species account for 5-10% of all ICU-acquired bloodstream infections, with mortality rates ranging from 40% to 60% despite appropriate antifungal therapy (Wisplinghoff et al., 2014; Weiner-Lastinger et al., 2020; Kwon et al., 2021). In patients with severe influenza or COVID-19-associated pneumonia, the incidence of invasive pulmonary aspergillosis can reach 19-30%, contributing significantly to poor outcomes (Schauwvlieghe et al., 2018; Verweij et al., 2020). These infections not only escalate mortality but also prolong mechanical ventilation duration, extend ICU and hospital stays by an average of 10–15 days, and substantially increase healthcare costs (Bassetti and Bouza, 2017).

The pathophysiology of IFI in sepsis patients involves a complex interplay of immune dysfunction, epithelial barrier disruption, and microbial ecological shifts. Sepsis-induced immunoparalysis—characterized by impaired neutrophil function, reduced antigen presentation, and T-cell exhaustion—creates a permissive environment for fungal invasion and dissemination (Lionakis and Levitz, 2018; Netea et al., 2020). The lung epithelium, already damaged by severe pneumonia, loses its integrity as a physical barrier, facilitating fungal spore germination and tissue invasion. Concurrently, broad-spectrum antibiotic use disrupts the normal microbiome, eliminating bacterial competition and promoting fungal colonization and overgrowth (Wu and Segal, 2018). Furthermore, the hyperinflammatory state in sepsis leads to endothelial damage and microthrombosis, which can trap fungal elements in small vessels, promoting tissue ischemia and creating niches for fungal proliferation (Ince et al., 2018). Despite these recognized risk factors, early diagnosis of IFI remains notoriously challenging in the ICU setting. Clinical signs are nonspecific and often masked by the underlying critical illness, while conventional culture-based methods suffer from low sensitivity (approximately 40% for invasive candidiasis) and significant time delays (Martin-Loeches et al., 2025). Biomarkers such as β-D-glucan and galactomannan have improved diagnostic capabilities but are not universally available, may yield false positives, and lack utility for screening in unselected populations (Ullmann et al., 2018).

Several prognostic scores exist for general sepsis outcomes, with the Sequential Organ Failure Assessment (SOFA) score being widely used for organ dysfunction quantification (Singer et al., 2016). However, these generic scores lack specificity for predicting fungal infections. While some IFI prediction models have been developed, they have limited applicability in sepsis patients with severe pneumonia, who represent a distinct pathophysiological subgroup (Bassetti et al., 2018). To address this critical gap, we aimed to develop and validate a specialized predictive nomogram that integrates readily available clinical and laboratory parameters to estimate individual IFI risk in sepsis patients with severe pneumonia. Such a tool could facilitate early identification of high-risk patients, enabling timely diagnostic evaluation and preemptive antifungal therapy, ultimately improving clinical outcomes in this vulnerable population.

## 2 Materials and methods

### 2.1 Study design

This retrospective cohort study was conducted at Shaanxi Provincial People’s Hospital, a tertiary care academic medical center, between January 2020 and October 2025. The study protocol was reviewed and approved by the Institutional Review Board of Shaanxi Provincial People’s Hospital (Approval No: 2025R082). To assess the generalizability of the nomogram, an independent external validation cohort was retrospectively collected from Xi’an No. 3 Hospital, a separate tertiary care center, between January 2023 and October 2025. The same inclusion and exclusion criteria, IFI diagnostic definitions, and variable collection protocols were applied.

### 2.2 Study population

We screened all consecutive adult patients (aged ≥18 years) admitted to the ICU with a primary diagnosis of sepsis and severe pneumonia. Exclusion criteria were rigorously applied to minimize confounding and ensure cohort homogeneity. Patients were excluded if they met any of the following conditions: (1) < 18 years old; (2) The length of stay in ICU was less than 24 hours; (3) Missing clinical information. After applying the exclusion criteria (including “Missing clinical information”), the final analytical dataset (n = 1,890) contained no missing values for any variable. Accordingly, no imputation methods were applied.

### 2.3 Definition

The diagnosis of sepsis was established according to the Sepsis-3 criteria, requiring a suspected or documented infection and an acute increase of ≥2 points in the SOFA score (Singer et al., 2016). Severe pneumonia was defined based on the 2019 American Thoracic Society/Infectious Diseases Society of America (ATS/IDSA) guidelines (Metlay et al., 2019), requiring the presence of at least one major criterion (septic shock with need for vasopressors or respiratory failure requiring mechanical ventilation) or three or more minor criteria (respiratory rate ≥30 breaths/min, PaO_2_/FiO_2_ ratio ≤250, multilobar infiltrates, confusion/disorientation, uremia, leukopenia, hypothermia, or hypotension requiring aggressive fluid resuscitation).

### 2.4 Diagnostic criteria for IFI

Given that the classic EORTC/MSG criteria were originally developed for immunocompromised cancer patients, we adapted the 2020 revised definitions to our ICU setting with the following specifications (Donnelly et al., 2020).

#### 2.4.1 Classification of diagnostic certainty

Each suspected IFI episode was retrospectively classified as proven, probable, or possible based on integration of host factors, radiological findings, and mycological evidence. Only proven and probable cases were included as IFI outcomes; possible cases were excluded to minimize false positives.

#### 2.4.2 Host factors (adapted for ICU sepsis patients)

In addition to classic EORTC/MSG host factors (neutropenia, hematologic malignancy, allogeneic stem cell transplantation, solid organ transplantation, prolonged corticosteroid use, T−cell immunosuppressants), we considered sepsis−associated immunoparalysis as an adjunct factor. However, final diagnosis relied on objective mycological evidence.

#### 2.4.3 Radiological criteria

For suspected invasive pulmonary aspergillosis (IPA), chest computed tomography (CT) findings were classified as: 1) Highly suggestive: halo sign, air−crescent sign, cavitary lesion, or hypodense sign. 2) Non−specific: nodule, consolidation, ground−glass opacity. Only patients with highly suggestive CT patterns were considered for probable IPA.

#### 2.4.4 Mycological methods

The following diagnostic tests were performed as clinically indicated: 1) Fungal culture: Blood, bronchoalveolar lavage (BAL), sputum, pleural fluid, or tissue specimens were cultured on Sabouraud dextrose agar; positive cultures were identified by matrix−assisted laser desorption/ionization time−of−flight mass spectrometry (MALDI−TOF MS). 2) Galactomannan (GM): Serum GM index ≥0.7 or BAL GM index ≥0.5 was considered positive (Platelia Aspergillus EIA, Bio−Rad). 3) β−D−glucan (BDG): Serum BDG ≥80 pg/mL was considered positive (Fungitell assay, Associates of Cape Cod), used only as adjunctive evidence. 4) Aspergillus PCR: Where available, positive PCR (as per European Aspergillus PCR Initiative guidelines) was accepted as mycological evidence. 5) Histopathology: Tissue biopsies showing invasive hyphae or yeast forms were considered gold standard for proven IFI.

#### 2.4.5 Specific definitions for major IFI entities

1) Proven invasive candidiasis: Histopathological demonstration of yeast in deep tissue OR positive blood culture for Candida species in a patient with clinical signs of sepsis and no other identifiable source. Candidemia was always considered proven if the blood culture was obtained from a percutaneous venipuncture (not from a central line that could reflect colonization). 2) Probable invasive candidiasis: Not applicable per EORTC/MSG; we only included proven cases to avoid overestimation. 3) Proven IPA: Histopathological evidence of hyphae with tissue invasion OR positive culture from a normally sterile site (e.g., pleural fluid) with compatible radiology. 4) Probable IPA: Presence of host factor + compatible CT pattern (halo sign, air−crescent sign, cavity) + mycological evidence (positive BAL GM ≥0.5, serum GM ≥0.7, or positive BAL culture for Aspergillus). 5) Other IFIs (e.g., mucormycosis, fusariosis, cryptococcosis) were diagnosed according to EORTC/MSG criteria using species−specific tests.

#### 2.4.6 Distinguishing colonization from invasive infection

##### 2.4.6.1 Candida colonization

A positive culture from a non−sterile site (sputum, urine, skin, or central line tip) without clinical signs of systemic infection and without histopathological evidence was considered colonization and excluded. 2) Aspergillus colonization: Isolation of Aspergillus from sputum or BAL without compatible CT pattern or GM positivity was considered colonization. 3) To further minimize misclassification, we applied a quantitative approach: for Candida, only blood cultures or deep−tissue cultures were accepted as invasive; for Aspergillus, only BAL or tissue cultures accompanied by GM positivity or typical CT signs were accepted.

#### 2.4.7 Fungal species identification and nomenclature

All fungal isolates were identified to species level using MALDI−TOF MS. For Candida species, we adopted the current taxonomic nomenclature: Candida albicans, Candida parapsilosis, Candida tropicalis, Nakaseomyces glabratus (formerly Candida glabrata), Clavispora lusitaniae (formerly Candida lusitaniae), and Meyerozyma guilliermondii (formerly Candida guilliermondii). Species distribution in the IFI cohort is reported in **Supplementary Table 1**.

#### 2.4.8 Diagnostic workflow and quality control

All potential IFI cases were independently reviewed by two infectious disease specialists (blinded to outcomes). Disagreements were resolved by consensus with a third specialist. Cases with missing key data (e.g., no mycological test, no CT) were excluded.

### 2.5 Data collection and variables

Trained research personnel, blinded to the study objectives, extracted comprehensive data from electronic medical records using a standardized data collection form. Collected variables included: (1) Demographics and Baseline Characteristics: Age, gender, body mass index (BMI).

(2) Vital Signs on ICU Admission: Temperature, respiratory rate (RR), heart rate (HR), mean arterial pressure (MAP). (3) Comorbidities: Hypertension, diabetes mellitus, acute respiratory distress syndrome (ARDS), respiratory failure (RF), chronic obstructive pulmonary disease (COPD), acute-on-chronic liver failure (ACFL), cirrhosis, septic shock, acute kidney injury (AKI), chronic kidney disease (CKD). (4) Laboratory Parameters: All laboratory tests were performed on samples collected within 24 hours of ICU admission. This included coagulation profiles [prothrombin activity (PTA), thrombin time (TT), international normalized ratio (INR), prothrombin time (PT), activated partial thromboplastin time (APTT), fibrinogen (FIB), D-dimer (D-D), fibrinogen degradation products (FDP)], liver and renal function tests [albumin, globulin, bilirubin, alkaline phosphatase (ALP), aspartate aminotransferase (AST), alanine aminotransferase (ALT), blood urea nitrogen (BUN), creatinine (Cr), uric acid (UA), cystatin-C], inflammatory markers [white blood cell count (WBC), neutrophil count (NEUT), lymphocyte count, monocyte count, C-reactive protein (CRP), procalcitonin (PCT)], and other hematological parameters [platelet count (PLT), hemoglobin, red blood cell count (RBC), red cell distribution width-coefficient of variation (RDW-CV), red cell distribution width-standard deviation (RDW-SD), mean platelet volume (MPV)], as well as lactate, glucose, and total cholesterol (TC). (5) Clinical Scores and Outcomes: The SOFA score was calculated based on the worst values within the first 24 hours of ICU admission. The primary outcome was the occurrence of IFI during the ICU stay. Secondary outcomes included hospital length of stay (LOS), use of mechanical ventilation, and requirement for continuous renal replacement therapy (CRRT).

### 2.6 ICU-related and immunosuppression variables

In addition to the variables described above, we systematically extracted data on the following factors known to influence IFI risk in critically ill patients: (1) Corticosteroid use: defined as administration of any systemic corticosteroid at a prednisone-equivalent dose ≥5 mg/day for ≥3 consecutive days within 7 days before ICU admission or during ICU stay. (2) Immunosuppression: defined as presence of any of the following within 3 months prior to ICU admission — chemotherapy, solid organ or hematopoietic stem cell transplantation, chronic immunosuppressive therapy (e.g., calcineurin inhibitors, methotrexate, azathioprine, mycophenolate mofetil, anti-TNF agents), or HIV infection with CD4 count <200 cells/μl. (3) Broad-spectrum antibiotic use: use of carbapenems, third/fourth-generation cephalosporins, piperacillin-tazobactam, vancomycin, or linezolid for ≥48 hours before IFI diagnosis. (4) Antifungal prophylaxis: systemic administration of fluconazole, echinocandins, amphotericin B formulations, voriconazole, or posaconazole before IFI diagnosis. (5) Central venous catheter (CVC): presence of a CVC during ICU stay, and its duration (days). (6) Mechanical ventilation duration: total days of invasive mechanical ventilation.

### 2.7 Statistical analysis

The total study population was randomly divided into a training set (75%) for model development and a validation set (25%) for internal validation, using a computer-generated random number sequence. Baseline characteristics were compared between the two sets using the Mann-Whitney U test for continuous variables (presented as medians with interquartile ranges) and the Chi-square test or Fisher’s exact test for categorical variables (presented as counts and percentages). Variable selection for the prediction model was a two-step process. First, univariate logistic regression was performed to identify variables associated with IFI (with a liberal *P* < 0.10 considered for further analysis). Second, to handle multicollinearity and enhance model generalizability, the Least Absolute Shrinkage and Selection Operator (LASSO) regression method was employed. The optimal tuning parameter (lambda) was selected via 10-fold cross-validation using the ‘one-standard-error’ rule (lambda.1se), which favors a more parsimonious model. Variables with non-zero coefficients from the LASSO regression were then entered into a multivariate logistic regression model to identify independent predictors and estimate their odds ratios (ORs) with 95% confidence intervals (CIs). A two-tailed *P* < 0.05 was considered statistically significant in the final multivariate model. The final model was presented as a nomogram for individualized risk prediction. Model performance was evaluated in terms of discrimination, calibration, and clinical utility. Discrimination, the ability to distinguish between patients with and without IFI, was assessed using the area under the receiver operating characteristic curve (AUC). Calibration, the agreement between predicted probabilities and observed outcomes, was evaluated with calibration plots and the Hosmer-Lemeshow test. Clinical utility was quantified using decision curve analysis (DCA), which estimates the net benefit across a range of threshold probabilities. The SOFA score was selected as a comparator due to its documented independent association with IFI risk in critically ill ICU patients and its established role as a benchmark in prediction model studies (Li et al., 2018; Yu et al., 2025).

All statistical analyses were performed using R software (version 4.2.1) and SPSS Statistics (version 26.0). A two-tailed *P* < 0.05 was considered statistically significant.

## 3 Results

### 3.1 Baseline characteristics of the study population

**Figure 1:**
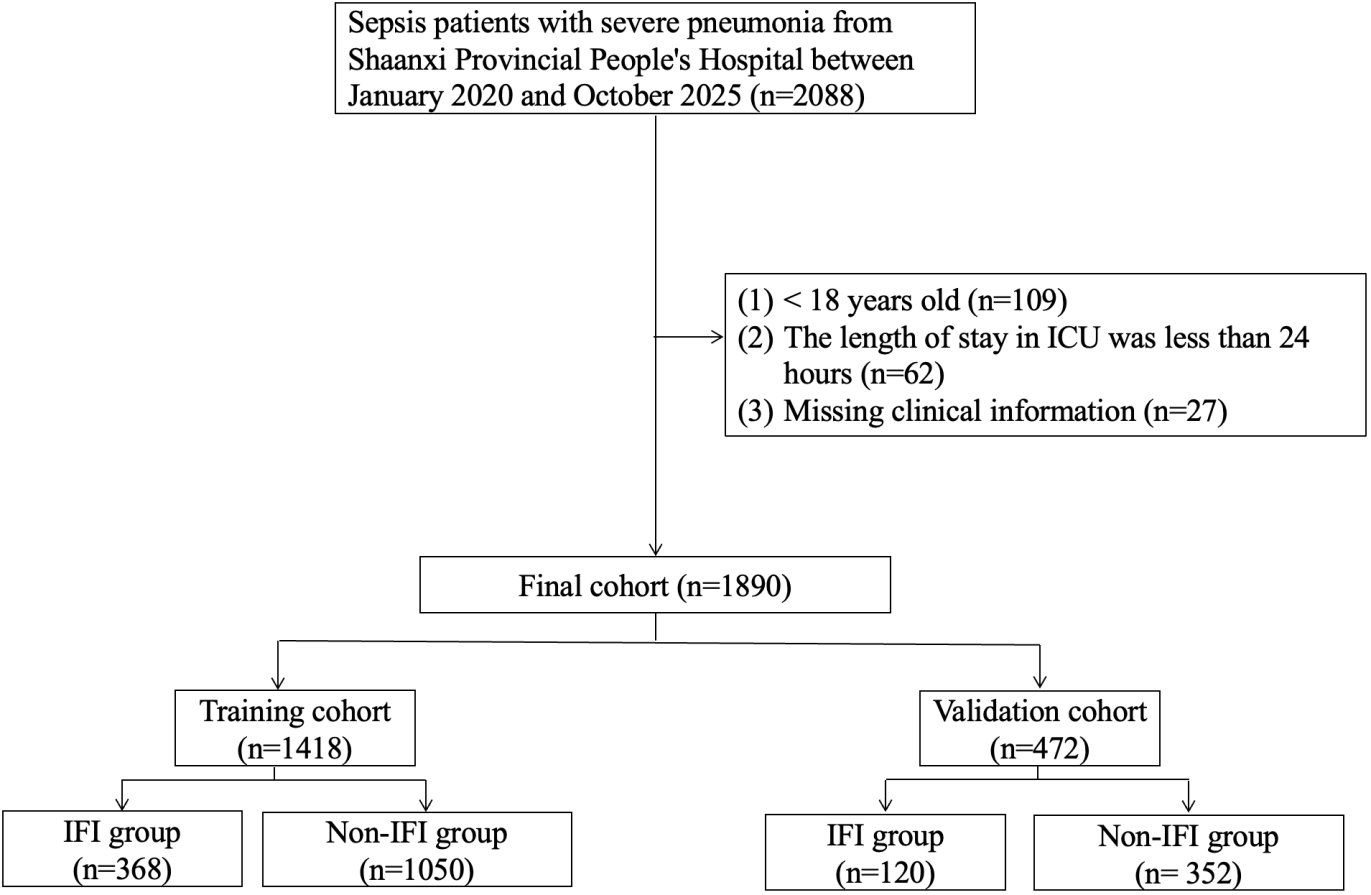
The flowchart of patient selection. invasive fungal infections (IFI).

A total of 1,890 septic patients with severe pneumonia were included in this study, of whom 488 (25.8%) developed IFI. The cohort was randomly divided into a training set (n=1,418) for model development and a validation set (n=472) for internal validation (**Figure 1**). The baseline characteristics, encompassing demographics, vital signs, comorbidities, and laboratory parameters, were well-balanced between the training and validation cohorts (all *P* > 0.05), confirming the comparability of the two datasets for subsequent model development and validation (**Table 1**).

**Table 1:** Baseline characteristics of sepsis patient with severe pneumonia.

Variable	Total (n =1890)	Training (n=1418)	Validation (n=472)	P value
IFI, n (%)				0.868
No	1402 (74.2)	1050 (74)	352 (74.6)	
Yes	488 (25.8)	368 (26)	120 (25.4)	
Age (years)	63 (51, 73)	62 (51, 73)	63 (51, 73)	0.925
Gender, n (%)				0.896
Female	1040 (55.0)	782 (55.1)	258 (54.7)	
Male	850 (45.0)	636 (44.9)	214 (45.3)	
Vital signs				
T(°C)	36.6 (36.3, 37.0)	36.3 (36.6, 37.0)	36.6 (36.3, 37.0)	0.645
RR (bpm)	20 (19, 23)	20 (19, 23)	20 (18, 23)	0.324
HR (bpm)	93 (78, 109)	93 (78, 109)	92 (78, 108)	0.359
MAP (mmHg)	131(117, 148)	131 (117, 149)	131 (119, 147)	0.731
Comorbidities				
Hypertension, n (%)				0.970
No	1270 (67.2)	952 (67.1)	318 (67.4)	
Yes	620 (32.8)	466 (32.9)	154 (32.6)	
Diabetes, n (%)				0.548
No	1351 (71.5)	1008 (71.1)	343 (72.7)	
Yes	539 (28.5)	410 (28.9)	129 (27.3)	
ARDS, n (%)				0.532
No	1611 (85.2)	1204 (84.9)	407 (86.2)	
Yes	279 (14.8)	214 (15.1)	65 (13.8)	
RF, n (%)				0.691
No	1345 (71.2)	1013 (71.4)	332 (70.3)	
Yes	545 (28.8)	405 (28.6)	140 (29.7)	
COPD, n (%)				0.340
No	1779 (94.1)	1330 (93.8)	449 (95.1)	
Yes	111 (5.9)	88 (6.2)	23 (4.9)	
ACFL, n (%)				1
No	1745 (92.3)	1309 (92.3)	436 (92.4)	
Yes	145 (7.7)	109 (7.7)	36 (7.6)	
Cirrhosis, n (%)				0.701
No	1695 (89.7)	1269 (89.5)	426 (90.3)	
Yes	195 (10.3)	149 (10.5)	46 (9.7)	
Septic shock, n (%)				0.627
No	1321 (69.8)	994 (70.1)	327 (69.3)	
Yes	569 (30.1)	424 (29.9)	145 (30.7)	
AKI, n (%)				0.857
No	1521 (80.5)	1143 (80.6)	378 (80.1)	
Yes	369 (19.5)	275 (19.4)	94 (19.9)	
CKD, n (%)				0.794
No	1666 (88.1)	1246 (87.9)	420 (89)	
Yes	224 (11.9)	172 (12.1)	52 (11)	
Laboratory test				
PTA (%)	78 (65, 89)	79 (66, 89)	77 (65, 88)	0.536
TT (S)	17.0 (15.8, 18.7)	17.1 (15.9, 18.8)	16.9 (15.7, 18.4)	0.101
INR	1.43 (1.29, 1.67)	1.42 (1.29, 1.66)	1.44 (1.29, 1.70)	0.486
FDP (mg/L)	11.3 (5.9, 22.5)	11.3 (5.8, 22.6)	11.2 (6.6, 21.9)	0.908
D-D (mg/L)	5.2 (2.5, 11.1)	5.2 (2.5, 11.2)	5.1 (2.6, 10.5)	0.754
FIB (g/L)	4.31 (2.81, 5.78)	4.21 (2.78, 5.75)	4.46 (2.95, 5.87)	0.196
APTT (S)	45.3 (38.5, 53.3)	45.3 (38.4, 53.4)	45.4 (38.6, 52.6)	0.937
PT (S)	16.6 (14.8, 19.3)	16.6 (14.7, 19.3)	16.7 (14.9, 19.3)	0.729
Lactate (mmol/L)	1.86 (1.20, 3.00)	1.90 (1.26, 3.00)	1.80 (1.13, 2.90)	0.098
Globulin (g/L)	25.3 (22.2, 29.7)	25.4 (22.1, 29.8)	25.3 (22.2, 29.6)	0.833
Albumin (g/L)	28.8 (25.2, 33.1)	28.8 (25.1, 33.0)	29.1 (25.5, 33.4)	0.229
Bilirubin (mg/dl)	17.7 (10.7, 36.7)	17.8 (10.6, 36.9)	17.5 (10.8, 35.5)	0.858
ALP	97.0 (68.0,147.0)	96.0 (68.0, 146.0)	92.0 (68.0, 151.8)	0.585
AST (U/L)	34.0 (22.0, 73.0)	34.0 (21.0, 72.0)	33.0 (23.0, 75.8)	0.502
ALT (U/L)	29.0 (17.0, 59.0)	29.0 (17.0, 59.0)	29.0 (17.3, 60.0)	0.487
Glucose (mmol/L)	6.97 (5.28, 10.00)	6.94 (5.29, 10.07)	7.08 (5.21, 9.79)	0.956
WBC (× 109/L)	7.97 (5.98, 14.85)	7.82 (5.98, 14.38)	8.49 (6.04, 15.59)	0.092
Monocyte (×109/L)	0.40 (0.22, 0.69)	0.40 (0.22, 0.69)	0.40 (0.24, 0.70)	0.441
RDW-CV	14.3 (13.3, 16.0)	14.3 (13.3, 15.9)	14.4 (13.3, 16.0)	0.634
RDW-SD	48.1 (44.1,54.5)	48.3 (44.2, 54.6)	48.0 (44.0, 54.2)	0.674
MPV (fL)	10.9 (9.9, 12.1)	10.9 (9.9, 12.1)	10.9 (9.9, 12.1)	0.919
NEUT (×109/L)	7.7 (4.3, 13.1)	7.6 (4.2, 12.9)	7.9 (4.6, 14.0)	0.140
Lymphocyte (×109/L)	0.65 (0.37, 1.07)	0.65 (0.37, 1.07)	0.65 (0.38, 1.05)	0.908
PLT (× 109/L)	126 (64, 205)	126 (64, 207)	127 (63, 201)	0.914
Hemoglobin (g/L)	101 (84, 121)	101 (83, 121)	102 (86, 121)	0.443
RBC (×10¹²/L)	3.35 (2.73, 3.99)	3.34 (2.70, 3.97)	3.41 (2.81, 4.05)	0.185
PCT (ng/ml)	2.00 (0.52, 12.93)	1.95 (0.51, 12.40)	2.20 (0.59, 13.44)	0.226
CRP (mg/L)	76.8 (19.9, 152.8)	75.7 (19.4, 151.1)	80.6 (21.8, 154.4)	0.544
UA (μmol/L)	298 (194, 417)	301 (195, 421)	289 (187, 406)	0.210
TC (mmol/L)	2.77 (1.95, 3.69)	2.77 (1.92, 3.67)	2.78 (1.97, 3.74)	0.742
Cystatin-C (mg/L)	1.44 (1.03, 2.50)	1.46 (1.03, 2.55)	1.43 (1.03, 2.33)	0.486
BUN (mmol/L)	9.5 (5.7,17.1)	9.5 (5.7, 17.2)	9.6 (5.7, 16.6)	0.801
Cr (μmol/L)	84.0 (55.0, 181.5)	87.0 (55.0, 184.0)	80.0 (54.0, 178.0)	0.315
SOFA	7 (6, 9)	7 (6, 9)	7 (6, 9)	0.361
Hospital LOS (days)	15 (9, 26)	15 (9, 26)	15 (9, 24)	0.545
CRRT, n (%)				0.988
No	1419 (75.1)	1064 (75.0)	355 (75.2)	
Yes	471 (24.9)	354 (25.0)	117 (24.8)	
Mechanical ventilation, n (%)				0.056
No	1243 (65.8)	915 (64.5)	328 (69.5)	
Yes	647 (34.2)	503 (35.5)	144 (30.5)	
Corticosteroid use				0.445
No	1,566 (82.86)	1,169 (82.44)	397 (84.11)	
Yes	324 (17.14)	249 (17.56)	75 (15.89)	
Immunosuppression				0.914
YES	451 (23.86)	337 (23.77)	114 (24.15)	
NO	1,439 (76.14)	1,081 (76.23)	358 (75.85)	
Broad spectrum antibiotic				0.202
YES	935 (49.47)	714 (50.35)	221 (46.82)	
NO	955 (50.53)	704 (49.65)	251 (53.18)	
Antifungal prophylaxis				0.611
No	1,823 (96.46)	1,370 (96.61)	453 (95.97)	
Yes	67 (3.54)	48 (3.39)	19 (4.03)	
CVC duration days	2.00 (1.00, 5.00)	2.00 (1.00, 5.00)	2.00 (1.00, 4.00)	0.486
MV duration days	0.00 (0.00, 3.00)	0.00 (0.00, 3.00)	0.00 (0.00, 3.00)	0.076

invasive fungal infections (IFI), respiratory rate (RR), heart rate (HR), mean arterial pressure (MAP), acute respiratory distress syndrome (ARDS), respiratory failure (RF), chronic obstructive pulmonary disease (COPD), Acute-on-chronic liver failure (ACFL), chronic kidney disease (CKD), acute kidney injury (AKI), prothrombin activity (PTA), international normalized ratio (INR), prothrombin time (PT), activated partial thromboplastin time (APTT), alkaline phosphatase (ALP), aspartate aminotransferase (AST), alanine aminotransferase (ALT), D-dimer (D-D), fibrinogen degradation products (FDP), fibrinogen (FIB), uric acid (UA), blood urea nitrogen (BUN), creatinine (Cr), white blood cell count (WBC), platelet count (PLT), C-reactive protein (CRP), procalcitonin (PCT), red cell distribution width-coefficient of variation (RDW-CV), red cell distribution width-standard deviation (RDW-SD), mean platelet volume (MPV), neutrophil count (NEUT), red blood cell (RBC), lymphocyte count, total cholesterol (TC), Sequential Organ Failure Assessment (SOFA), continuous renal replacement therapy (RRT).

### 3.2 Predictor selection using LASSO regression

**Figure 2:**
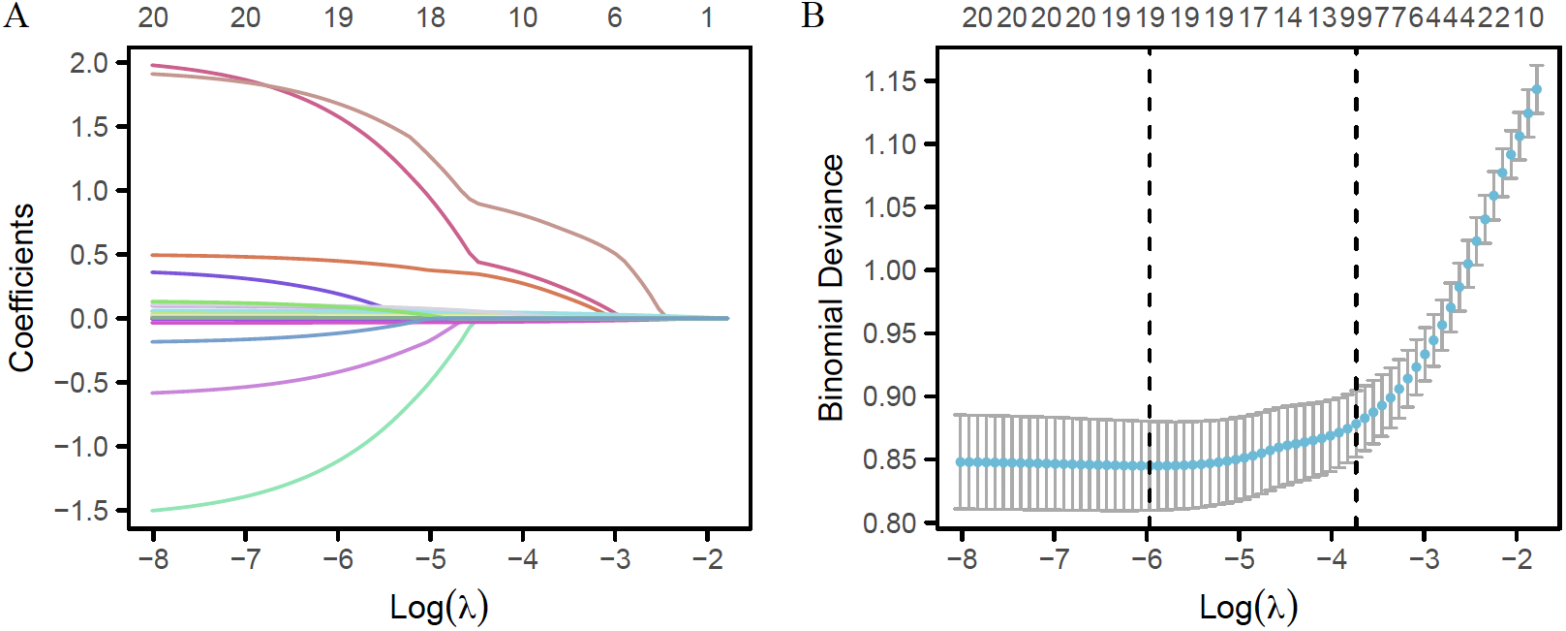
Identification of clinical predictors via LASSO regression. **(A)** Coefficient profiles of features across the regularization path. **(B)** Ten-fold cross-validation for optimal lambda selection (lambda.1se) using the one-standard-error rule, yielding the final parsimonious predictor set.

Univariate logistic regression analysis initially identified numerous variables significantly associated with IFI occurrence (**Table 2**). To refine the most relevant predictors and mitigate multicollinearity, the LASSO regression method was employed. The coefficient profiles illustrate the contraction of feature coefficients as the penalty parameter (lambda) increases (**Figure 2A**). The optimal lambda (lambda.1se) was selected via ten-fold cross-validation, applying the one-standard-error rule to favor a parsimonious model without substantially compromising predictive accuracy, resulting in a final set of 9 non-zero coefficient predictors (**Figure 2B**).

**Table 2:** Univariate analysis of predictive biomarkers of IFI in the training cohort.

Variables	OR	95% CI	P value
Age (years)	1.001	0.994-1.009	0.701
Gender	0.928	0.730-1.178	0.538
Vital signs			
T(°C)	1.238	1.080-1.418	0.002
RR (bpm)	1.039	1.020-1.059	< 0.001
HR (bpm)	1.005	1.000-1.010	0.075
MAP (mmHg)	1.004	0.999-1.009	0.154
Comorbidities			
Hypertension	1.035	0.804-1.331	0.790
Diabetes	2.196	1.709-2.821	< 0.001
Ascites	1.013	0.688-1.491	0.948
ACFL	4.508	3.019-6.733	< 0.001
ARDS	1.763	1.295-2.401	< 0.001
COPD	3.985	2.567-6.177	< 0.001
RF	2.145	1.669-2.758	< 0.001
Septic shock	1.577	1.226-2.027	< 0.001
CKD	0.912	0.630-1.320	0.625
AKI	0.945	0.698-1.280	0.717
Laboratory test			
PTA (%)	0.968	0.961-0.974	< 0.001
TT (S)	1.004	1.000-1.008	0.066
INR	1.364	1.137-1.637	< 0.001
FDP (mg/L)	1.001	0.999-1.003	0.204
D-D (mg/L)	1.044	1.033-1.055	< 0.001
FIB (g/L)	1.095	1.039-1.153	<0.001
APTT (S)	1.076	1.063-1.089	< 0.001
PT (S)	1.066	1.046-1.086	< 0.001
Lactate (mmol/L)	1.066	1.011-1.123	0.017
Globulin (g/L)	1.013	0.998-1.027	0.093
Albumin (g/L)	0.987	0.969-1.006	0.188
Bilirubin (mg/dl)	0.999	0.998-1.000	0.219
ALP (U/L)	1.000	0.999-1.001	0.524
AST (U/L)	1.000	1.000-1.000	0.485
ALT (U/L)	1.000	1.000-1.000	0.675
Glucose (mmol/L)	1.010	0.986-1.034	0.411
WBC (× 109/L)	1.018	1.005-1.031	0.009
Monocyte (×109/L)	1.012	0.979-1.047	0.473
RDW-CV	0.981	0.939-1.025	0.391
RDW-SD	0.990	0.978-1.002	0.101
NEUT (×109/L)	1.000	0.985-1.015	0.980
Lymphocyte (×109/L)	1.003	0.989-1.018	0.636
PLT (× 109/L)	1.000	0.999-1.001	0.836
MPV (fL)	0.930	0.960-1.006	0.069
Hemoglobin (g/L)	0.999	0.994-1.003	0.555
RBC (×10¹²/L)	0.998	0.873-1.141	0.974
PCT (ng/ml)	1.004	1.000-1.008	0.049
CRP (mg/L)	1.004	1.002-1.005	< 0.001
UA (μmol/L)	1.000	0.999-1.001	0.930
TC (mmol/L)	0.913	0.843-0.989	0.026
Cystatin-C (mg/L)	1.039	0.960-1.126	0.343
BUN (mmol/L)	1.005	0.994-1.017	0.334
Cr (μmol/L)	1.000	1.000-1.001	0.672
SOFA	1.418	1.343-1.497	< 0.001
Hospital LOS (days)	1.005	0.999-1.012	0.118
CRRT	1.147	0.876-1.503	0.319
Mechanical ventilation	1.144	0.894-1.463	0.284
Corticosteroid use	1.448	1.076-1.950	0.015
Immunosuppression	1.727	1.324-2.251	< 0.001
Broad spectrum antibiotic	1.103	0.870-1.399	0.417
Antifungal prophylaxis	1.446	0.784-2.667	0.238
CVC duration days	1.025	0.981-1.071	0.272
MV duration days	1.033	0.989-1.079	0.139

Although we added the two ICU-related and immunosuppression variables (corticosteroid use and immunosuppression) to the LASSO regression together with all previously described clinical and laboratory parameters, none of these two variables were selected (all coefficients shrunk to zero) under the lambda.1se criterion. Variables that could represent consequences rather than antecedents of IFI (e.g., hospital length of stay, mechanical ventilation, CRRT) were not selected by LASSO (coefficients shrunk to zero) and thus did not enter the final model. All eight retained predictors are baseline characteristics assessed at or before ICU admission, eliminating concerns about reverse causality. The final parsimonious model therefore retained only the original eight predictors (RR, diabetes, RF, ACFL, PTA, D-dimer, APTT, lactate).

invasive fungal infections (IFI), respiratory rate (RR), heart rate (HR), mean arterial pressure (MAP), acute respiratory distress syndrome (ARDS), respiratory failure (RF), chronic obstructive pulmonary disease (COPD), Acute-on-chronic liver failure (ACFL), chronic kidney disease (CKD), acute kidney injury (AKI), prothrombin activity (PTA), international normalized ratio (INR), prothrombin time (PT), activated partial thromboplastin time (APTT), alkaline phosphatase (ALP), aspartate aminotransferase (AST), alanine aminotransferase (ALT), D-dimer (D-D), fibrinogen degradation products (FDP), fibrinogen (FIB), uric acid (UA), blood urea nitrogen (BUN), creatinine (Cr), white blood cell count (WBC), platelet count (PLT), C-reactive protein (CRP), procalcitonin (PCT), red cell distribution width-coefficient of variation (RDW-CV), red cell distribution width-standard deviation (RDW-SD), mean platelet volume (MPV), neutrophil count (NEUT), red blood cell (RBC), lymphocyte count, total cholesterol (TC), Sequential Organ Failure Assessment (SOFA), continuous renal replacement therapy (RRT).

### 3.3 Independent predictors and nomogram construction

**Figure 3:**
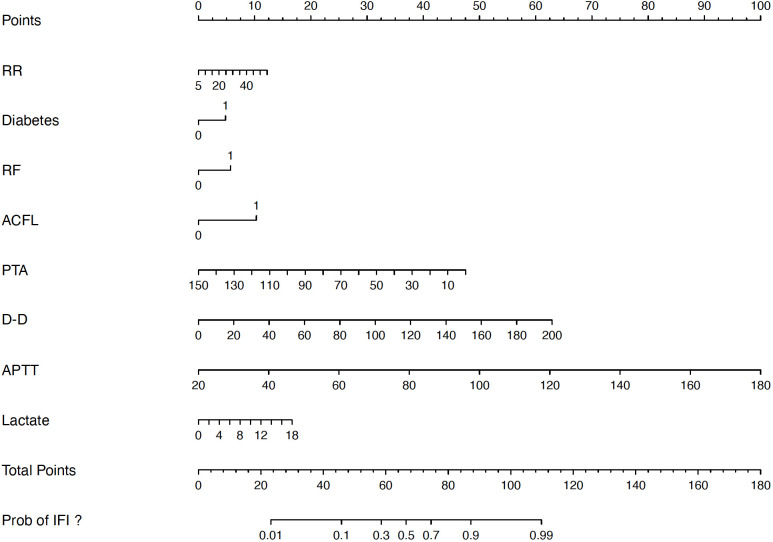
A prognostic nomogram to predict the risk of IFI in sepsis patients with severe pneumonia.

Variables selected by the LASSO regression were subsequently incorporated into a multivariate logistic regression analysis to identify independent predictors. Eight factors were ultimately retained (**Table 3**): RR (OR: 1.026), Diabetes (OR: 1.669), RF (OR: 1.834), ACFL (OR: 2.987), PTA (PTA, OR: 0.967), D-dimer (OR: 1.034), APTT (OR: 1.069), and Lactate (OR: 1.103). These independent predictors, reflecting respiratory, metabolic, hepatic, coagulation, and perfusion dimensions, were integrated to construct a user-friendly prognostic nomogram for individualized IFI risk estimation (**Figure 3**).

**Table 3:** Multivariate logistic regression analysis of independent predictors of IFI.

Variables	β	SE	Wald	P-value	OR (95% Cl)
RR	0.026	0.011	5.150	0.023	1.026 (1.004-1.050)
Diabetes	0.512	0.159	10.363	0.001	1.669 (1.222-2.280)
RF	0.607	0.162	14.065	<0.001	1.834 (1.336-2.519)
ACFL	1.094	0.254	18.531	<0.001	2.987 (1.815-4.917)
PTA	-0.034	0.004	74.305	< 0.001	0.967 (0.960, 0.974)
D-D	0.033	0.006	30.291	< 0.001	1.034 (1.022, 1.046)
APTT	0.066	0.007	99.432	< 0.001	1.069 (1.055, 1.083)
Lactate	0.098	0.033	8.960	0.003	1.103 (1.034, 1.176)
Constant	-3.473	0.512	46.058	< 0.001	0.031

invasive fungal infections (IFI), respiratory rate (RR), respiratory failure (RF), Acute-on-chronic liver failure (ACFL), prothrombin activity (PTA), activated partial thromboplastin time (APTT), D-dimer (D-D).

### 3.4 Collinearity diagnostics and correlation heatmap analysis

To formally assess potential multicollinearity among the candidate variables, we calculated the variance inflation factor (VIF) and tolerance statistic for all variables that achieved univariate significance (*P* < 0.05). As shown in **Supplementary Table 2**, all VIF values were below the conventional threshold of 5, indicating that severe multicollinearity was not present.

Correlation heatmap analysis (**Supplementary Figure 1**) showed that both corticosteroid use and immunosuppression were significantly associated with multiple organ failure-related variables (diabetes, ARDS, RF, COPD, ACFL, cirrhosis, and septic shock; all *P* < 0.001). These substantial overlaps in clinical information explain why LASSO, under the parsimonious lambda.1se criterion, shrank the coefficients of corticosteroid use and immunosuppression to zero while retaining the original eight predictors that directly capture end−organ dysfunction and coagulopathy.

### 3.5 Predictive performance, calibration, and clinical utility

**Figure 4:**
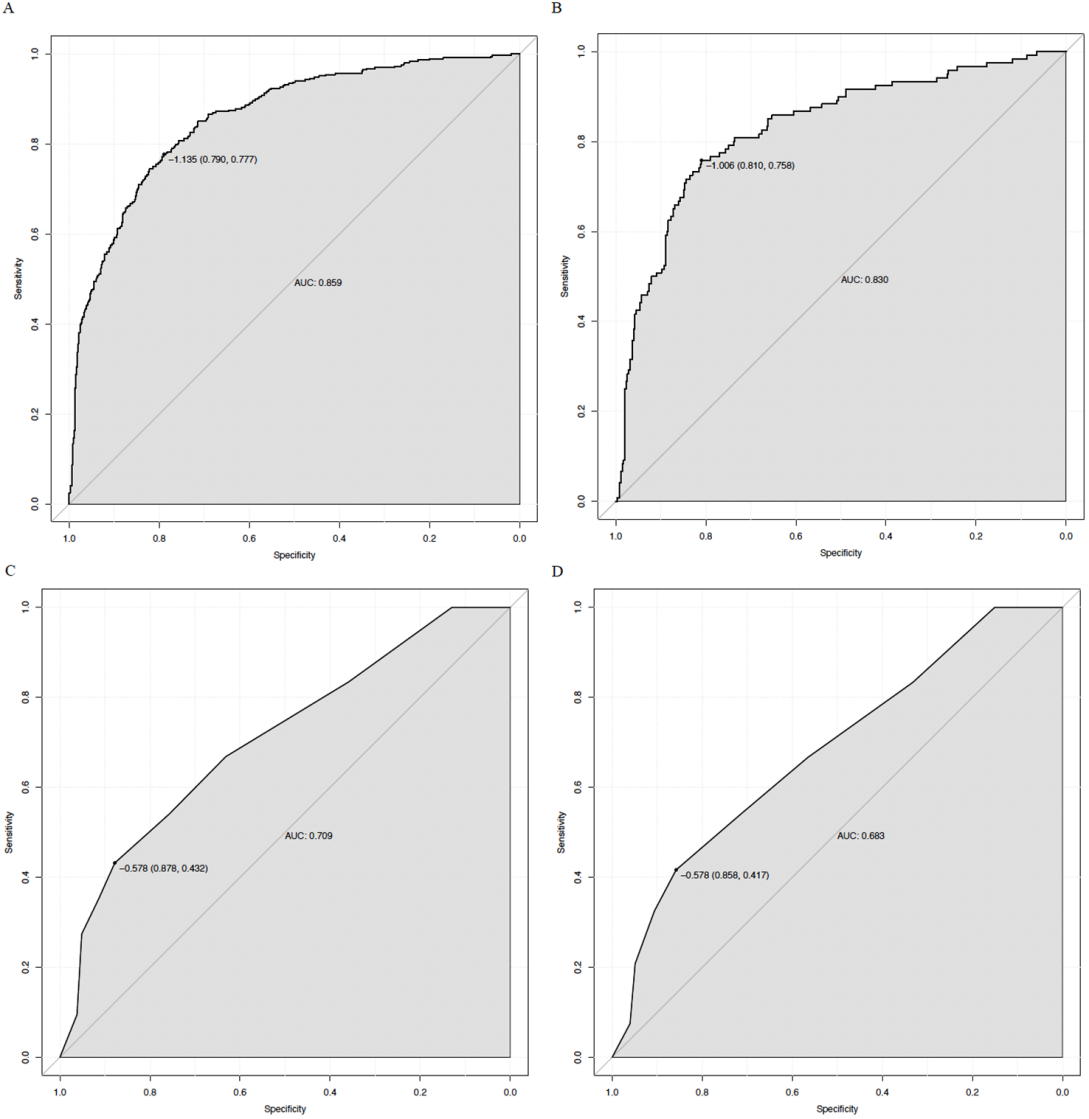
Comparative assessment of the nomogram and SOFA score for IFI prediction. **(A)** Nomogram in the training set (AUC = 0.859). **(B)** Nomogram in the internal validation set (AUC = 0.830). **(C)** SOFA score in the training set (AUC = 0.709). **(D)** SOFA score in the validation set (AUC = 0.683).

**Figure 5:**
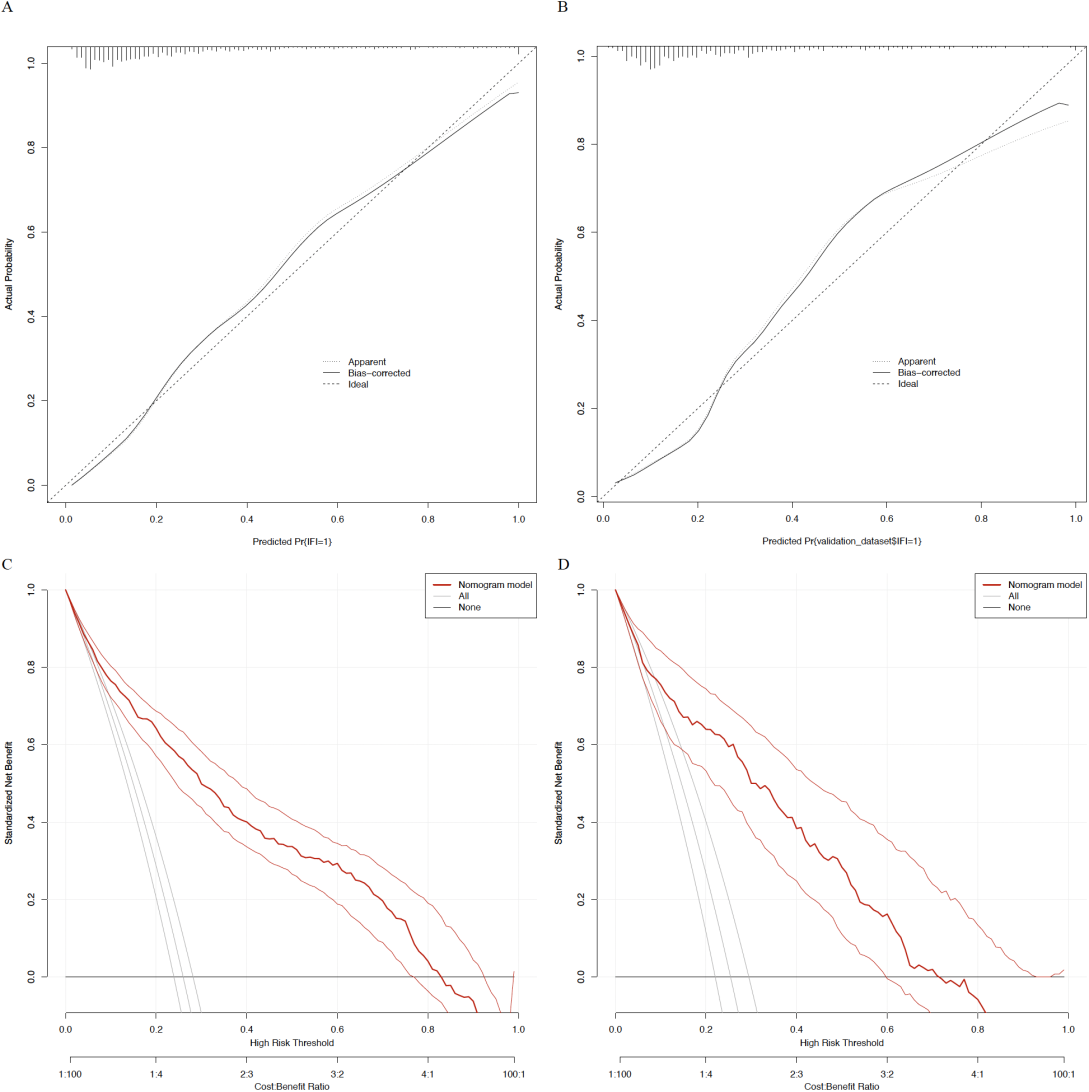
Evaluation of model calibration and clinical utility for the IFI prediction nomogram. **(A, B)** Calibration curves depict the concordance between predicted probabilities and actual outcomes. The diagonal dashed line represents the ideal calibration. **(C, D)** Decision curve analysis (DCA) estimates the net benefit of using the nomogram for clinical decision-making against different intervention thresholds.

The predictive accuracy of the newly developed nomogram was evaluated using ROC curve analysis. The model demonstrated excellent discriminative ability, with AUC values of 0.859 in the training cohort and 0.830 in the validation cohort (**Figure 4**). In contrast, the SOFA score yielded significantly lower AUCs of 0.709 and 0.624 in the respective cohorts. The calibration curves showed strong agreement between predicted probabilities and observed outcomes in both sets (**Figures 5A, B**). Furthermore, DCA confirmed that the application of this nomogram provides a superior net benefit across a wide range of threshold probabilities, highlighting its substantial potential for guiding clinical decision-making (**Figures 5C, D**).

receiver operating characteristic (ROC), area under the receiver operating characteristics curve (AUC), sequential organ failure assessment (SOFA).

### 3.6 The external validation using the prediction model

**Figure 6:**
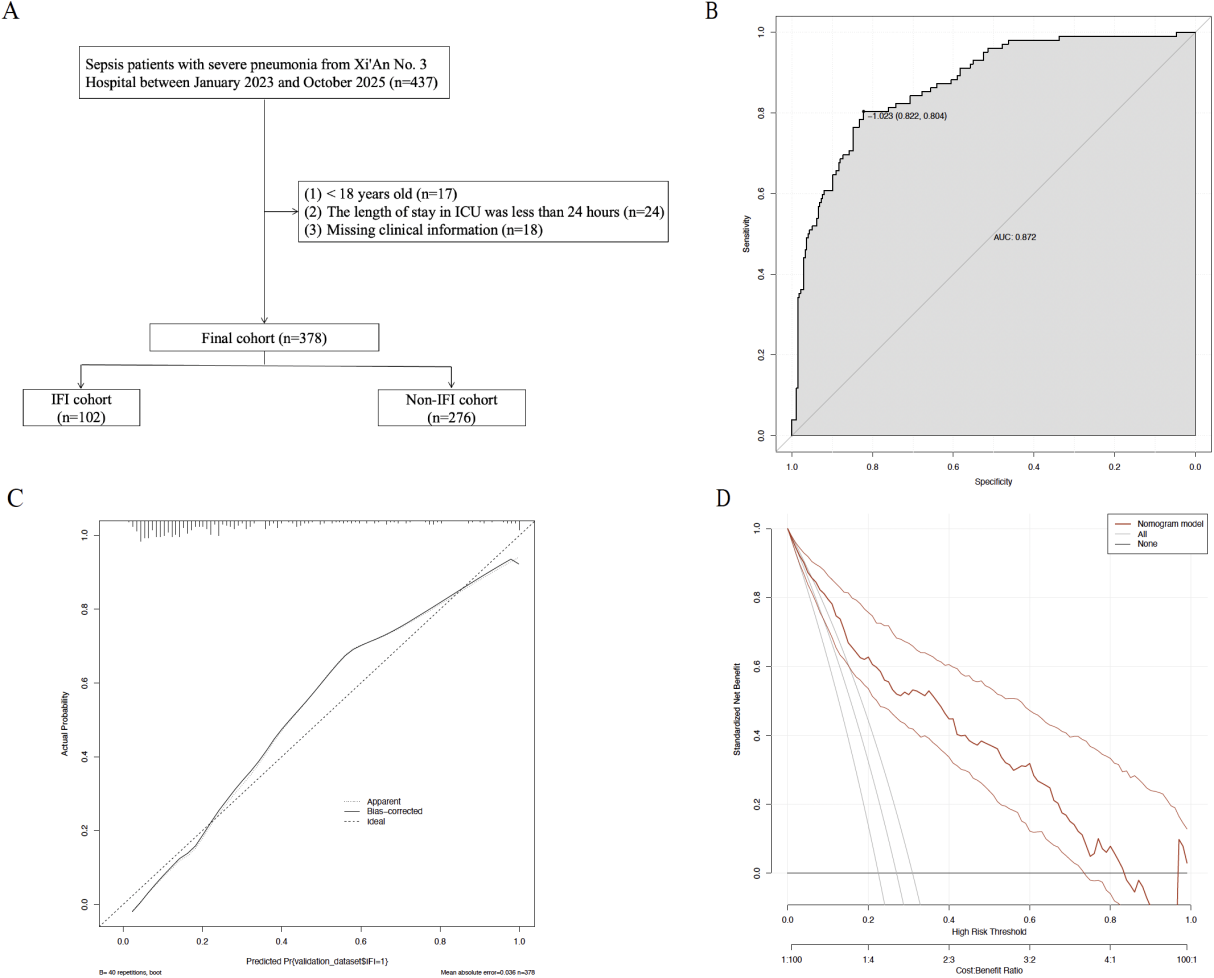
External validation of the predictive nomogram. **(A)** Flowchart illustrating patient selection at Xi’an No. 3 Hospital. **(B)** Receiver operating characteristic (ROC) curve of the nomogram for IFI prediction in the external validation cohort. **(C)** Calibration curve demonstrating agreement between predicted probabilities and observed outcomes. **(D)** Decision curve analysis (DCA) assessing the clinical utility of the nomogram in the external validation set.

An independent external validation cohort comprising 378 sepsis patients with severe pneumonia from Xi’an No. 3 Hospital was used to further evaluate the nomogram. As shown in **Figure 6**, the nomogram maintained excellent discriminative ability, achieving an AUC of 0.872. The calibration curve demonstrated good agreement between predicted risks and observed IFI rates, and the DCA confirmed a positive net benefit over a wide range of threshold probabilities, indicating robust clinical utility in an independent setting.

### 3.7 Sensitivity analyses of the model including corticosteroid use and immunosuppression

To evaluate the independent predictive value of corticosteroid use and immunosuppression, we forced these two variables into the multivariate logistic regression together with the eight original predictors (RR, diabetes, RF, ACFL, PTA, D−dimer, APTT, lactate). **Supplementary Figure 2** shows the extended nomogram, and **Supplementary Figure 3** presents its ROC curves, calibration curves, and DCA.

In the training cohort, the extended model achieved an AUC of 0.861 versus 0.859 for the original model. In the internal validation cohort, AUC was 0.831 versus 0.830. Calibration and DCA curves were nearly superimposable, and the extended model did not improve net benefit. These results confirm that adding the two variables does not provide incremental predictive value.

## 4 Discussion

In this large, retrospective cohort study, we developed and internally validated a novel, user-friendly nomogram for predicting the risk of IFI in a high-risk, yet specific, population of sepsis patients with severe pneumonia in the ICU. The model incorporates eight readily obtainable clinical and laboratory parameters: respiratory rate, diabetes, respiratory failure, acute-on-chronic liver failure, prothrombin activity, D-dimer, activated partial thromboplastin time, and lactate. It demonstrated robust performance, with excellent discrimination (AUC 0.859 in training, 0.830 in validation), satisfactory calibration, and a positive net benefit across a wide range of clinical decision thresholds, significantly outperforming the commonly used SOFA score.

### 4.1 Pathophysiological basis of the selected predictors

The predictive variables in our nomogram span multiple organ systems and collectively delineate a clinical phenotype at high risk for fungal invasion. Elevated respiratory rate and respiratory failure reflect severe lung injury and often necessitate mechanical ventilation, which breaches anatomical barriers and facilitates fungal spore germination and tissue invasion (Feldman et al., 2020). Additionally, diabetes impairs neutrophil function and T−lymphocyte activity, while advanced glycation end−products may promote fungal angioinvasion (de Souza Ferreira et al., 2012; Yamagishi et al., 2015; Jafar et al., 2016; Rhee and Kim, 2018). The strong association with acute−on−chronic liver failure (ACFL) highlights the liver’s role in host defense: hepatic dysfunction reduces complement synthesis and impairs clearance of fungal antigens, and elevated free iron secondary to liver failure further supports fungal growth (Liu et al., 2015; Schaarschmidt et al., 2018; Berndt et al., 2024; Vandendriessche et al., 2024). Markers of coagulopathy (decreased PTA, elevated D−dimer, prolonged APTT) indicate endothelial injury and microthrombosis, creating niches for fungal proliferation (Neves et al., 2017; Tang et al., 2020; Hansen et al., 2021; Radotra and Challa, 2022). Finally, elevated lactate, a marker of tissue hypoperfusion, also exerts direct immunomodulatory effects that compound sepsis−induced immunoparalysis (Liu et al., 2024; Shukla et al., 2024; Zhang et al., 2025). Together, these variables capture a host response phenotype that integrates respiratory, metabolic, hepatic, coagulation, and perfusion dimensions.

### 4.2 Comparison with existing IFI prediction models

Several prediction models for IFI have been developed for ICU populations. The Candida score (León et al., 2006) assigns points for surgery, total parenteral nutrition, multifocal colonization, and severe sepsis/septic shock, with an AUC of approximately 0.70–0.72. However, it is designed specifically to distinguish colonization from invasive candidiasis in non−neutropenic patients, whereas our nomogram targets a broader IFI definition (including Aspergillus and other fungi) in sepsis patients with severe pneumonia, achieving superior discrimination (AUC 0.859 internal, 0.872 external). The model by Li et al. (2018) identified SOFA score, antibiotic treatment period, and positive Candida albicans culture from non−sterile sites as predictors for invasive fungal disease in critically ill patients. While their model shared the SOFA score as a common element, our nomogram includes distinct parameters (RR, diabetes, RF, ACFL, PTA, D−dimer, APTT, lactate) that directly capture the pathophysiological processes driving IFI susceptibility in this specific population. The IPA−GRRR−OH score (Friol et al., 2025) provides a dedicated prediction model for invasive pulmonary aspergillosis in immunocompromised patients with acute respiratory failure, achieving AUCs of 0.72 (derivation) and 0.85 (validation). However, it targets a narrower, immunocompromised subgroup and focuses exclusively on IPA, whereas our nomogram addresses both Candida and Aspergillus infections in a broader sepsis−severe pneumonia population. Compared to these tools, our nomogram offers several advantages: (I) it is specifically designed for sepsis patients with severe pneumonia—a high−risk population lacking validated models; (II) it uses readily available clinical and laboratory parameters; (III) it demonstrates excellent discrimination with external validation; and (IV) it provides a simple, user−friendly graphical tool for bedside risk assessment.

### 4.3 Clinical applicability and practical implementation

The strength of our nomogram lies in its simplicity and feasibility in routine ICU practice. All eight predictors are routinely collected or easily measured at ICU admission: respiratory rate and the diagnoses of respiratory failure and diabetes are part of standard clinical assessment; ACFL is a well−defined clinical entity; PTA, D−dimer, APTT, and lactate are part of standard coagulation and metabolic panels. No specialized fungal biomarkers (e.g., galactomannan, β−D−glucan) are required, making the tool applicable even in resource−limited settings where such tests are unavailable or results are delayed. The nomogram provides an individualised probability of IFI, which can guide pre−emptive diagnostic evaluation (e.g., early bronchoscopy with BAL culture or galactomannan testing) and risk−stratified antifungal prophylaxis. For example, a high predicted risk (>0.5) may prompt empirical antifungal therapy while awaiting culture results, whereas a low risk (<0.2) could support antifungal stewardship by discouraging unnecessary exposure. However, the nomogram is not intended to replace clinical judgment; it should be used as an adjunct to clinical assessment. External validation in an independent cohort (Xi’an No. 3 Hospital, AUC 0.872) supports its generalizability, but we acknowledge that calibration may vary across different healthcare systems with different baseline IFI incidences. Future implementation studies should assess whether integration of this nomogram into electronic health records or clinical decision support systems improves patient outcomes (e.g., reduced time to appropriate antifungal therapy, lower mortality).

### 4.4 Interpretation of ICU-related and immunosuppression variables

The SOFA score is primarily recognized as a tool for quantifying organ dysfunction and predicting mortality in septic patients (Rhee et al., 2017). Nevertheless, we selected SOFA as a comparator for two reasons. First, mounting evidence has established a direct association between the degree of organ failure—as captured by the SOFA score—and the risk of invasive fungal infections. A previous prediction model for invasive fungal disease in critically ill ICU patients identified SOFA score as an independent predictor (together with antibiotic treatment period and positive Candida albicans culture from non-sterile sites) (Li et al., 2018). The mechanistic basis for this association likely lies in sepsis−induced immunoparalysis: patients with multiple organ dysfunction syndrome (MODS) exhibit a counter−anti−inflammatory response syndrome (CARS) that profoundly impairs antifungal immunity, creating a permissive environment for fungal invasion (Hartemink et al., 2003). Second, in the field of predictive modeling, comparing a newly developed nomogram against established scoring systems such as SOFA represents a standard practice to demonstrate incremental predictive value. Numerous studies have benchmarked their nomograms against SOFA to show superior discrimination, and the combination of net reclassification improvement (NRI) and integrated discrimination improvement (IDI) with AUC comparison provides a robust framework for such evaluation (Yu et al., 2025). Therefore, while SOFA is not itself a direct predictor of infection, its ability to capture the severity of organ dysfunction makes it a clinically meaningful and literature−supported comparator for assessing our IFI nomogram. Importantly, our nomogram significantly outperformed SOFA (AUC 0.859 vs. 0.709 in training, and 0.830 vs. 0.683 in validation), underscoring its added clinical value beyond standard organ−failure assessment.

Although we systematically collected six additional risk factors (corticosteroid use, immunosuppression, broad-spectrum antibiotic use, antifungal prophylaxis, CVC duration, and MV duration), none of these variables were retained as independent predictors in the final LASSO−selected model. Several explanations may account for this observation. First, collinearity likely exists between these variables and the original predictors. The LASSO procedure tends to select the most parsimonious set of non−redundant predictors, favoring the original variables that directly capture end−organ dysfunction and coagulopathy. Second, low prevalence of certain exposures may have limited power: only 3.5% of patients received antifungal prophylaxis, and broad−spectrum antibiotic use (present in 49.5%) did not show a univariate association with IFI (P = 0.417). Third, the original eight−variable model already exhibited excellent discrimination (AUC 0.859), leaving little residual variation to be explained by additional variables. Therefore, our nomogram remains robust and parsimonious.

To address the concern regarding generalizability, we performed an external validation using an independent cohort from Xi’an No. 3 Hospital. The nomogram yielded an AUC of 0.872, calibration slope close to 1, and a favorable DCA curve, demonstrating that the model’s predictive accuracy and clinical utility are reproducible outside the derivation institution. These results support the potential applicability of the nomogram across different ICU settings, although further validation in geographically diverse populations and with prospective designs remains warranted.

### 4.5 Limitations

Several limitations should be acknowledged. First, the derivation cohort was from a single center, and although external validation was performed in one independent hospital, broader generalizability to other regions or healthcare systems requires further validation. Second, the retrospective design may introduce selection bias and residual confounding, despite rigorous exclusion criteria and adjustment for multiple variables. Third, detailed data on antimicrobial exposure (e.g., cumulative dose, combination therapy) were not available, which may have influenced fungal colonization and infection risk. Fourth, the EORTC/MSG criteria were originally developed for immunocompromised cancer patients and may not fully capture IFI in ICU sepsis patients; we adapted them but prospective validation of our diagnostic algorithm is needed. Fifth, the low incidence of antifungal prophylaxis (3.5%) and the lack of histopathological confirmation in most cases (only 7.8% had tissue biopsy) may have led to misclassification, although “possible” IFI cases were excluded and independent expert review was performed. Sixth, while we collected six ICU−related and immunosuppression variables, none entered the final LASSO model, partly due to collinearity and low event rates; their absence does not imply clinical unimportance but suggests their effects are captured by existing predictors. Finally, prospective implementation studies are needed to assess the real−world clinical utility and cost−effectiveness of the nomogram.

## 5 Conclusion

In conclusion, our nomogram represents a significant step forward in the personalized assessment of IFI risk in sepsis patients with severe pneumonia. By integrating readily available clinical parameters that reflect multiple aspects of the host-fungal interaction, it provides clinicians with a practical tool for identifying high-risk patients who might benefit from enhanced surveillance or preemptive therapy. Future research should focus not only on validating this model in external cohorts but also on exploring the mechanistic connections between these predictors and fungal pathogenesis, potentially revealing new targets for therapeutic intervention.

## Data Availability

The raw data supporting the conclusions of this article will be made available by the authors, without undue reservation.
