# Supplementary material for: Construction and validation of a predictive nomogram model for invasive fungal infections in sepsis patients with severe pneumonia in the ICU
Source: Front Cell Infect Microbiol. 2026 Jul 1;16:1854135. doi: 10.3389/fcimb.2026.1854135 (PMC13368662; doi:10.3389/fcimb.2026.1854135)
Supplement: Supplementary file 1 [file DataSheet1.pdf]

Supplementary Table S1. Microbiological characteristics of IFI cases (n=488)

Pathogen / Diagnostic method	Number of cases (n)	Proportion (%)
**Fungal species**		
Candida species (total)	312	63.9
Candida albicans	132	42.3 (within Candida)
Nakaseomyces glabratus (formerly *Candida glabrata*)	89	28.5
Candida parapsilosis	44	14.1
Candida tropicalis	34	10.9
Other *Candida* species	13	4.2
Aspergillus species (total)	146	29.9
Aspergillus fumigatus	118	80.8 (within Aspergillus)
Aspergillus flavus	15	10.3
Aspergillus niger	8	5.5
Other *Aspergillus* species	5	3.4
Other fungi (total)	30	6.2
Cryptococcus neoformans	12	40.0 (within other)
Rhizopus spp. (mucormycosis)	8	26.7
Fusarium spp.	6	20.0
Other	4	13.3
**Diagnostic method (any positive)**		
Blood culture (Candida)	278	57.0
BAL culture (Aspergillus)	98	20.1
Serum galactomannan (≥0.7)	102	20.9
BAL galactomannan (≥0.5)	76	15.6
Serum β-D-glucan (≥80 pg/mL)	213	43.6
Aspergillus PCR (BAL/serum)	45	9.2
Histopathology (tissue biopsy)	38	7.8
Diagnostic certainty		
Proven IFI	186	38.1
Probable IFI	302	61.9
Diagnostic method (any positive)		
Blood culture (Candida)	278	57.0
BAL culture (Aspergillus)	98	20.1
Serum galactomannan (≥0.7)	102	20.9
BAL galactomannan (≥0.5)	76	15.6
Serum β-D-glucan (≥80 pg/mL)	213	43.6
Aspergillus PCR (BAL/serum)	45	9.2
Histopathology (tissue biopsy)	38	7.8
**Diagnostic certainty**		
Proven IFI	186	38.1
Probable IFI	302	61.9

Note: Percentages for species subgroups are calculated within the respective group (e.g., within Candida, within Aspergillus, within other fungi). Percentages for diagnostic methods represent proportion of total IFI cases (n=488) that had at least one positive test of that type; a single case could contribute multiple positive tests. BAL = bronchoalveolar lavage; PCR = polymerase chain reaction.

Supplementary Table S2. Diagnostic methods for collinearity among variables

Variable	Tolerance statistic	VIF
Constant		
T	0.913	1.095
RR	0.883	1.133
Hypertension	0.890	1.123
Diabetes	0.631	1.584
ARDS	0.203	4.938
RF	0.255	3.922
COPD	0.295	3.392
ACFL	0.264	3.786
Cirrhosis	0.304	3.291
Septic shock	0.222	4.498
PTA	0.882	1.134
INR	0.797	1.255
D_D	0.935	1.069
FIB	0.668	1.498
APTT	0.810	1.234
PT	0.726	1.377
Lactate	0.980	1.020
CRP	0.709	1.411
WBC	0.976	1.025
Corticosteroid use	0.936	1.069
Immunosuppression	0.348	2.874

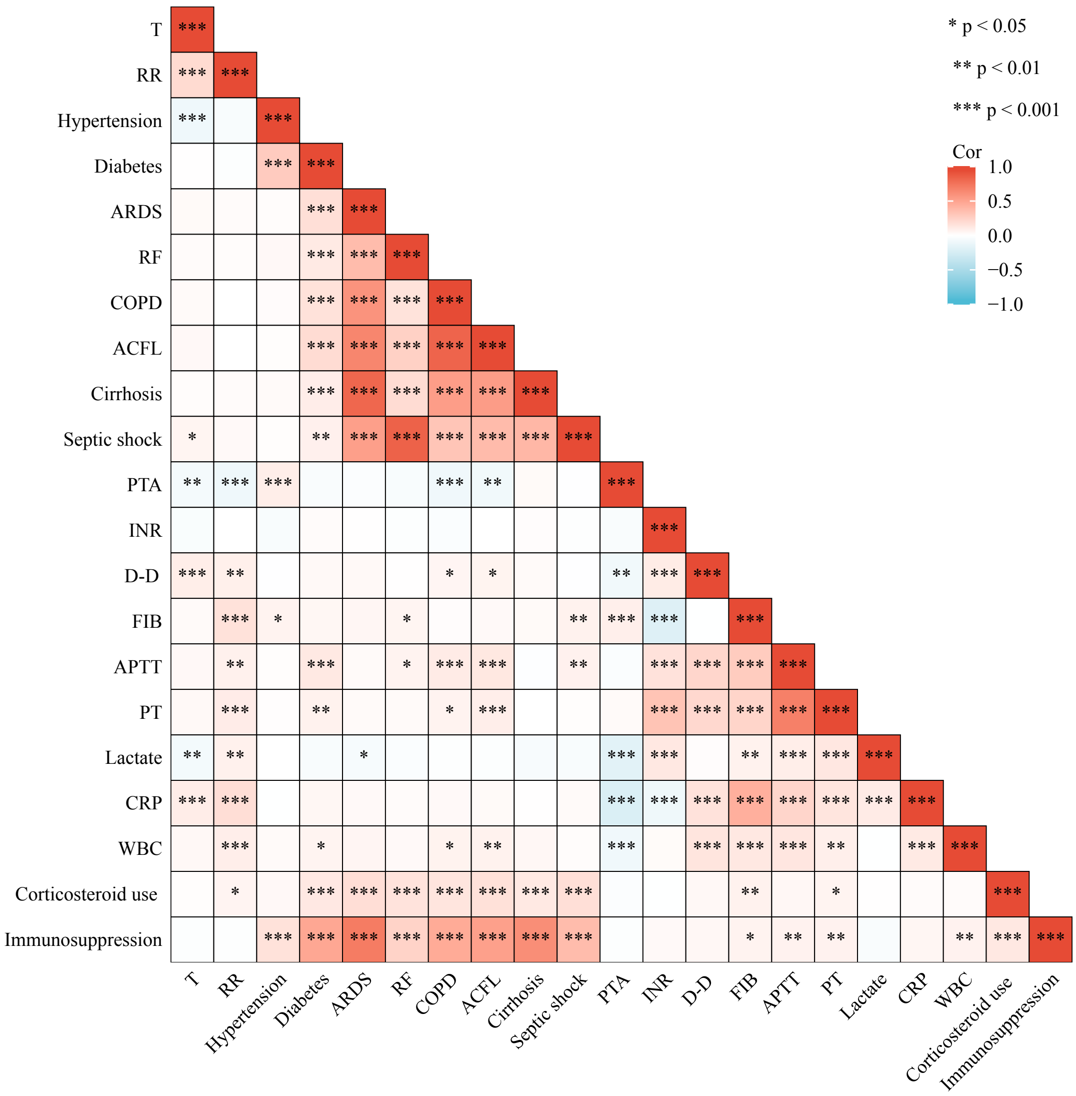

Supplementary Figure S1. Correlation heatmap analysis between variables

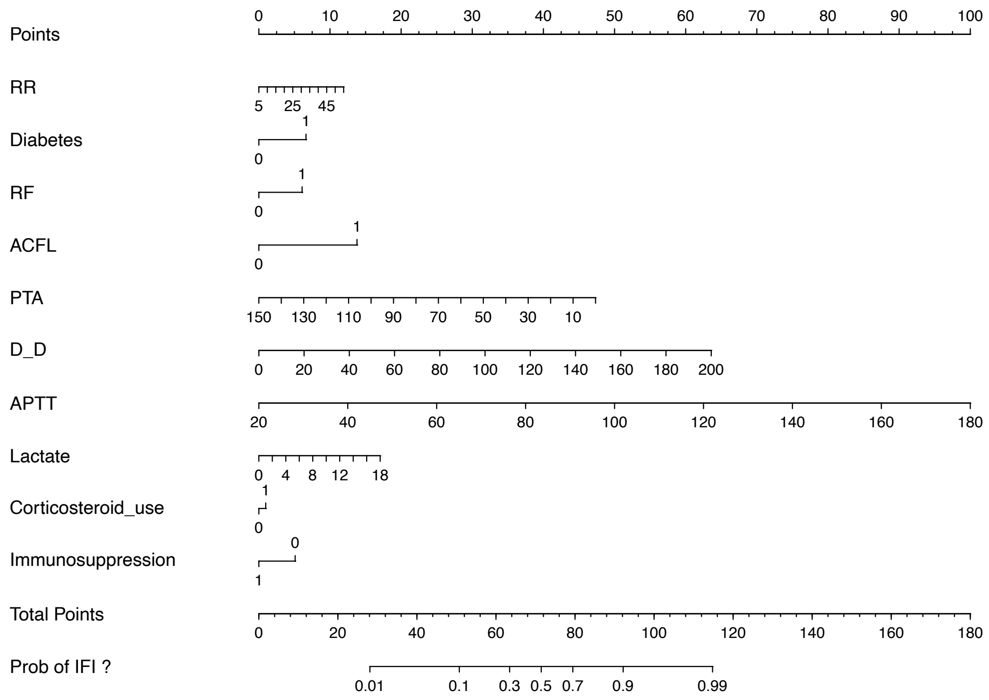

Figure S2. Nomogram of the extended model including corticosteroid use and immunosuppression for sensitivity analysis.

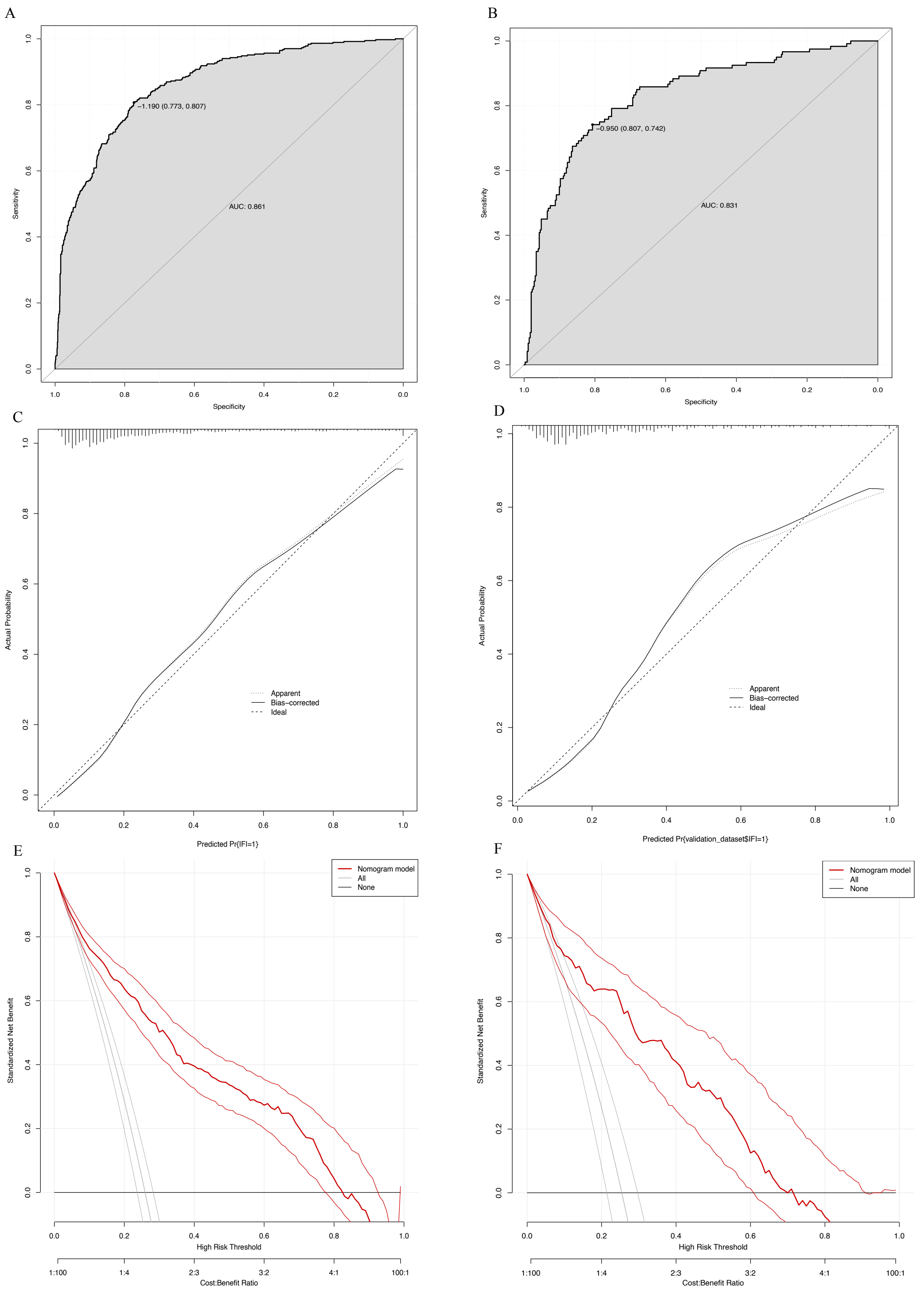

Figure S3. Discrimination, calibration, and clinical utility of the extended nomogram in the training and internal validation cohorts. (A) ROC curves. (B) Calibration curves. (C) Decision curve analysis.
